# A radiogenomics application for prognostic profiling of endometrial cancer

**DOI:** 10.1038/s42003-021-02894-5

**Published:** 2021-12-06

**Authors:** Erling A. Hoivik, Erlend Hodneland, Julie A. Dybvik, Kari S. Wagner-Larsen, Kristine E. Fasmer, Hege F. Berg, Mari K. Halle, Ingfrid S. Haldorsen, Camilla Krakstad

**Affiliations:** 1 Centre for Cancer Biomarkers, Department of Clinical Science, University of Bergen, Bergen, Norway; 2 Department of Obstetrics and Gynecology, Haukeland University Hospital, Bergen, Norway; 3 Mohn Medical Imaging and Visualization Centre (MMIV), Department of Radiology, Haukeland University Hospital, Bergen, Norway; 4 Section of Radiology, Department of Clinical Medicine, University of Bergen, Bergen, Norway

**Keywords:** Endometrial cancer, Cancer imaging

## Abstract

Prognostication is critical for accurate diagnosis and tailored treatment in endometrial cancer (EC). We employed radiogenomics to integrate preoperative magnetic resonance imaging (MRI, *n* = 487 patients) with histologic-, transcriptomic- and molecular biomarkers (*n* = 550 patients) aiming to identify aggressive tumor features in a study including 866 EC patients. Whole-volume tumor radiomic profiling from manually (radiologists) segmented tumors (*n* = 138 patients) yielded clusters identifying patients with high-risk histological features and poor survival. Radiomic profiling by a fully automated machine learning (ML)-based tumor segmentation algorithm (*n* = 336 patients) reproduced the same radiomic prognostic groups. From these radiomic risk-groups, an 11-gene high-risk signature was defined, and its prognostic role was reproduced in orthologous validation cohorts (*n* = 554 patients) and aligned with The Cancer Genome Atlas (TCGA) molecular class with poor survival (copy-number-high/p53-altered). We conclude that MRI-based integrated radiogenomics profiling provides refined tumor characterization that may aid in prognostication and guide future treatment strategies in EC.

## Abstract (web summary)

Hoivik, Hodneland and colleagues employ convoluted neural networks alongside radiogenomic biomarkers to profile endometrial tumors. They identify an 11-gene high-risk signature validated with retrospective patient data.

## Introduction

Endometrial cancer (EC) is the most prevalent cancer of the female reproductive tract in countries ranked within the highest tier of the human developmental index, with obesity being a strong predisposing factor^1,2^. While most EC patients have a favorable prognosis, identification of high-risk EC disease is essential to determine optimal surgical treatment and potential adjuvant therapies^3,4^. Preoperative risk stratification is based on endocervical curettage (biopsy) or endometrial pipelle sample often combined with preoperative imaging, such as pelvic magnetic resonance imaging (MRI) for local staging^3,5,6^. The increased use of advanced preoperative imaging and the introduction of novel machine learning techniques have opened for in-depth analyses of tumor characteristics relevant for diagnosis and prognostication. Radiomics is an emerging technique allowing tumor profiling by extracting quantitative information from medical images using mathematical descriptors and has been shown to predict clinical- and molecular tumor characteristics, survival, and response to treatment across various cancers^7,8^. Radiogenomics combines radiomics with genomic data to noninvasively determine underlying molecular characteristics^7–11^. Radiogenomics-based models reportedly predict genetic variants, i.e., mutations, microsatellite instability (MSI), gene expression, and tumor heterogeneity in non-small cell lung cancer and breast cancer, making this approach highly promising for developing personalized medicine^11–14^.

An increasing number of radiomics studies have linked radiomic profiling to prognosis in EC. Studies based on computed tomography (CT), MRI, and positron emission tomography (PET)-CT from single slice tumor segmentations^15–21^ and whole-volume tumor segmentations^14,22–25^ have identified radiomic signatures associated with high-risk features and poor prognosis^15–18,20,21^. Radiomic models predicting lymph node metastasis in EC have also been published^22–25^. Recently, whole-volume tumor radiomics from contrast-enhanced CT (CE-CT) was shown to predict high mutational burden in ECs, including The Cancer Genome Atlas (TCGA)-equivalent MSI tumors^14^. Similarly, a single-slice MRI radiomic prognostic index vector was recently linked to a specific gene expression signature that predicted poor outcome in EC^18^. Furthermore, deep-learning applications using MRI in EC have been used to derive valid automated tumor segmentations^26^ and reportedly provide promising models for automatic determination of myometrial depth^27,28^.

The aim of this study was to develop a novel radiogenomics approach using noninvasive, preoperative whole-volume tumor MRI for expedited radiomic based individual risk assessment and develop a corresponding prognostic gene expression signature in EC patients. Furthermore, we aimed to assess whether ML-based automated whole-volume tumor segmentations yield similar radiomic profiles that may be linked to the same gene expression signature.

## Results

### Unsupervised clustering of radiomic profiles identifies patients with high-risk clinico-pathological characteristics and poor outcome

**Fig. 1:**
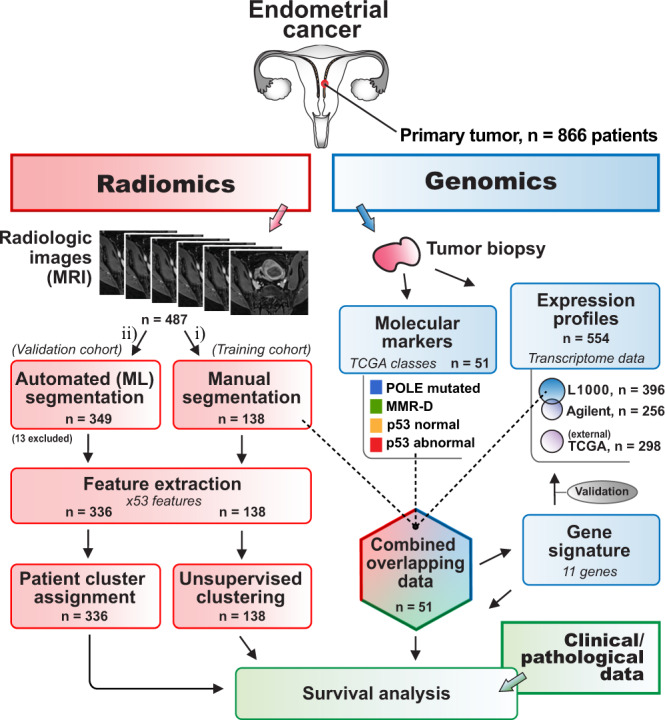
Radiogenomics approach in the current study of 866 endometrial cancer patients. Overview of the analytical approach integrating radiomics (red), genomics (blue), and clinical/pathological data (green). Preoperative MRI from 487 endometrial cancer patients were used for primary tumor segmentation using two approaches; (i) manual segmentation by radiologists (training cohort, 138 patients) and (ii) automated segmentation (using machine learning [ML] validation cohort, 349 patients, of which 13 patients were excluded due to failed automated tumor detection). Unsupervised clustering of extracted radiomic features in the manually segmented cohort (*n* = 138) yielded distinct radiomic clusters tested for differences in survival and clinico-pathological characteristics. Similarly, upon feature extraction, patients assigned to clusters by the automated segmentation approach (*n* = 336) were tested for survival differences. Resected tumors were profiled by transcriptome expression and analyzed for molecular markers. Transcriptome profiles were obtained by L1000 and Agilent expression array data (554 patients, 98 overlappings). For a subset of patients (hexagon, *n* = 51), MRI, expression profiles, and TCGA molecular classes were available, enabling the generation of a gene signature. The gene signature was validated in all transcriptome datasets, including the external TCGA RNA sequencing expression dataset (*n* = 298) and evaluated in survival analysis.

**Fig. 2:**
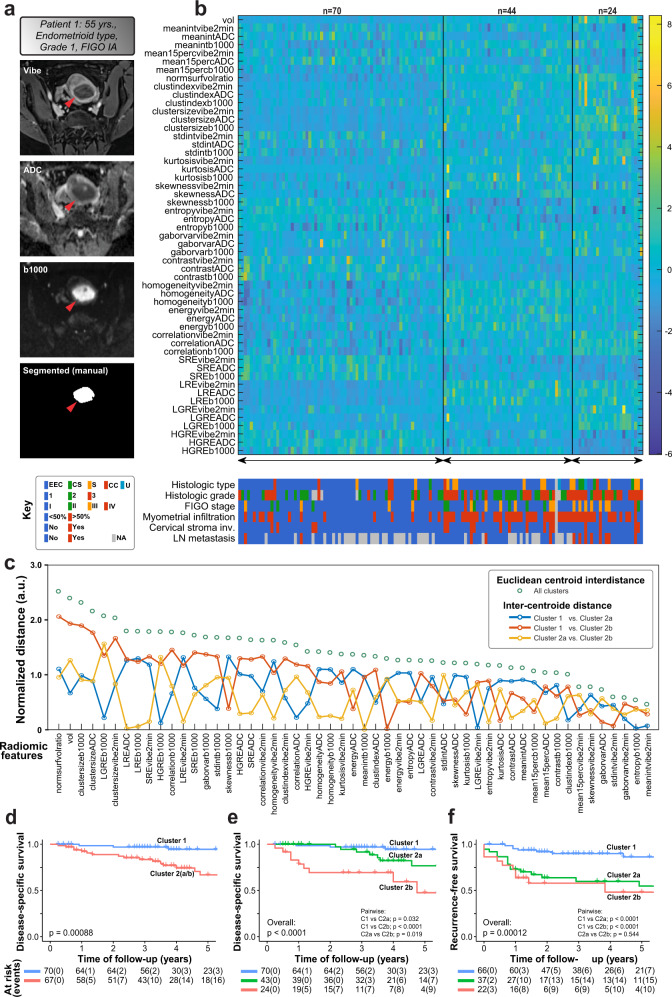
Unsupervised clustering of radiomic (3D) tumor features in 138 EC patients yields distinct clusters displaying different prevalence of high-risk features. **a** Preoperative pelvic MRI with manual tumor segmentation of the primary tumor (red arrows) of a patient with endometrioid type, histologic grade 1, and FIGO stage IA. The following MRI sequences were used for radiomic profiling of the manual segmentation-cohort; contrast-enhanced volumetric interpolated breath-hold examination (VIBE + C), apparent diffusion coefficient (ADC) map and diffusion-weighted sequence with b-value of 1000 (b1000) using the segmentation mask as that for VIBE + C. **b** Unsupervised clustering reveals three radiomic clusters with differences in clinico-pathological variables, reflecting differences in their risk-profiles. Patients in cluster 2a (*n* = 44) and cluster 2b (*n* = 24) more often had high-risk clinico-pathological features compared to patients in cluster 1 (*n* = 70). **c** Representing feature importance of the 53 derived radiomic features in terms of pairwise cluster centroid inter-distance (solid lines with open dots) and the aggregated value (open dots, “All clusters”; scaled for visualization). A large pairwise inter-distance indicates discriminating properties of large importance between clusters for a given texture feature. **d**–**f** Kaplan–Meier survival curves depicting significantly reduced disease-specific survival among radiomic clusters 1 and 2a/b combined (**d**), all three clusters (**e**), or by recurrence (**f**). The number of events in brackets. Histological types; EEC endometrioid, CS carcinosarcoma, S serous, CC clear cell, U undifferentiated.

A total of 866 EC patients were included in this study, of which 487 patients had available preoperative whole-volume tumor MRI and 554 patients had mRNA expression profiles and/or molecular markers. An outline of the approach to integrate radiomic, transcriptomic, molecular makers, and clinico-pathological data is displayed in Fig. 1. Fifty-three radiomic features were extracted from manually segmented primary tumors depicted at MRI in 138 EC patients. MRI sequences (VIBE + C, ADC, b1000) and the corresponding manual tumor segmentation mask used for radiomic tumor profiling is shown for one representative patient in Fig. 2a. A robust linear regression demonstrated a weak positive correlation between tumor volume and z-normalized “normsurfvolratio” (MATLAB, “robustfit”, *n* = 336, slope = 0.0090 ml^−1^, *p* = 0.0013). A similar curve fit for tumors with V > 1 ml yielded no significant correlation between z-normalized “normsurfvolratio” and tumor volume (MATLAB, “robustfit”, *n* = 288, slope = 0.0043 ml^−1^, *p* = 0.13), hence suggesting independency of tumor volume in larger tumors.

Unsupervised clustering of the radiomic features identified two distinct patient clusters; cluster 1 (*n* = 70) and cluster 2 (*n* = 68). Cluster 2 was characterized by higher values for “clusterindex”, “clustersize”, “homogeneity”, “energy”, “correlation”, and “LRE” and lower values for the radiomic features “contrast” and “SRE”, compared to cluster 1. Cluster 2 was subsequently re-clustered into subclusters 2a (*n* = 44) and 2b (*n* = 24) using the same unsupervised clustering algorithm; 2b had higher values for “LGRE” and “clustersize”, and lower values for “HGRE” compared to cluster 2a (Fig. 2b). Patients in clusters 2a/b more often presented high-risk clinico-pathological features including deep myometrial infiltration (>50%; *p* = 0.001), lymph node metastases (*p* = 0.020), high-grade histology (*p* = 0.001), non-endometrioid subtype (*p* = 0.014), and advanced FIGO stages (*p* = 0.006) (Fig. 2b, bottom panel; Table 1). Cluster 2b tumors were of higher grade (*p* = 0.050) and were more likely to be of non-endometrioid type (*p* = 0.050) compared to tumors of cluster 2a (Supplementary Table S1). Inter-centroid distance for all radiomic features indicated that no single radiomic feature was able to discriminate between the three clusters and that the interdependency across features was pronounced (Fig. 2c). When accounting for centroid inter-distance, two volume-related features (“normsurfvolratio” and “vol”) were ranked as the most important features describing the three clusters.

**Table 1:** Clinico-pathological variables in relation to the radiomic clusters based on the manually segmented tumors (*n* = 138).

Variable	Description	Radiomic clusters, *n* (%)		*p* value^a^
		Cluster 1 (*n* = 70)	Cluster 2a/b (*n* = 68)	
Age	<66	38 (57.6)	28 (42.4)	0.130
	≥66	32 (44.4)	40 (55.6)	
Histologic type	Endometrioid	63 (56.3)	49 (43.8)	0.014
	Non-endometrioid	7 (28.0)	18 (72.0)	
Histologic grade^b^	Grade 1–2	55 (63.2)	32 (36.8)	0.001
	Grade 3	5 (22.7)	17 (77.3)	
FIGO stage	I–II	66 (55.9)	52 (44.1)	0.006
	III–IV	4 (21.1)	15 (78.9)	
Myometrial infiltration	<50%	56 (75.7)	18 (24.3)	<0.001
	≥50%	14 (22.2)	49 (77.8)	
Lymph node metastasis	No	40 (48.8)	42 (51.2)	0.020
	Yes	2 (14.3)	12 (85.7)	
Ploidy	Diploid	24 (43.6)	31 (56.4)	0.424
	Aneuploidy	6 (31.6)	13 (68.4)	
ER protein expression^c^	High expression	27 (52.9)	24 (47.1)	0.059
	Low expression	8 (29.6)	19 (70.4)	
PR protein expression^c^	Positive	31 (52.5)	28 (47.5)	0.064
	Negative	5 (26.3)	14 (73.7)	
AR protein expression^c^	Positive	16 (51.6)	15 (48.4)	0.451
	Negative	12 (41.4)	17 (58.6)	

Missing data (numbers): Histologic type (1), Histologic grade (3), FIGO stage (1), Myometrial infiltration (1).

Not assessed (numbers): Lymph node metastasis (42), Ploidy (64), ER (60), PR (60), and AR (78).

Including one inoperable patient.

^a^ Calculated with Chi-Square test or Fischer’s exact test, as appropriate.

^b^ Endometrioid type only.

^c^ Protein levels by immunohistochemistry (IHC).

Patients in cluster 2 exhibited significantly reduced disease-specific survival (*p* < 0.001, Fig. 2d). Furthermore, patients in cluster 1, 2a, and 2b had significantly different disease-specific- and recurrence-free survival (overall *p* < 0.001; Fig. 2e, f) with the best outcome for patients in cluster 1, the intermediate outcome for patients in cluster 2a, and poorest outcome for patients in cluster 2b.

After dilation and erosion of the segmentation masks, only 14% (20/138) or 8% (11/138) of the cases changed clusters upon dilation and erosion, respectively. Furthermore, the finding that patients in the three clusters had significantly different survival was reproduced (*p* ≤ 0.001 for all; Supplementary Fig. S1).

### An 11-gene radiogenomic signature predicts aggressive EC phenotypes and poor survival

**Fig. 3:**
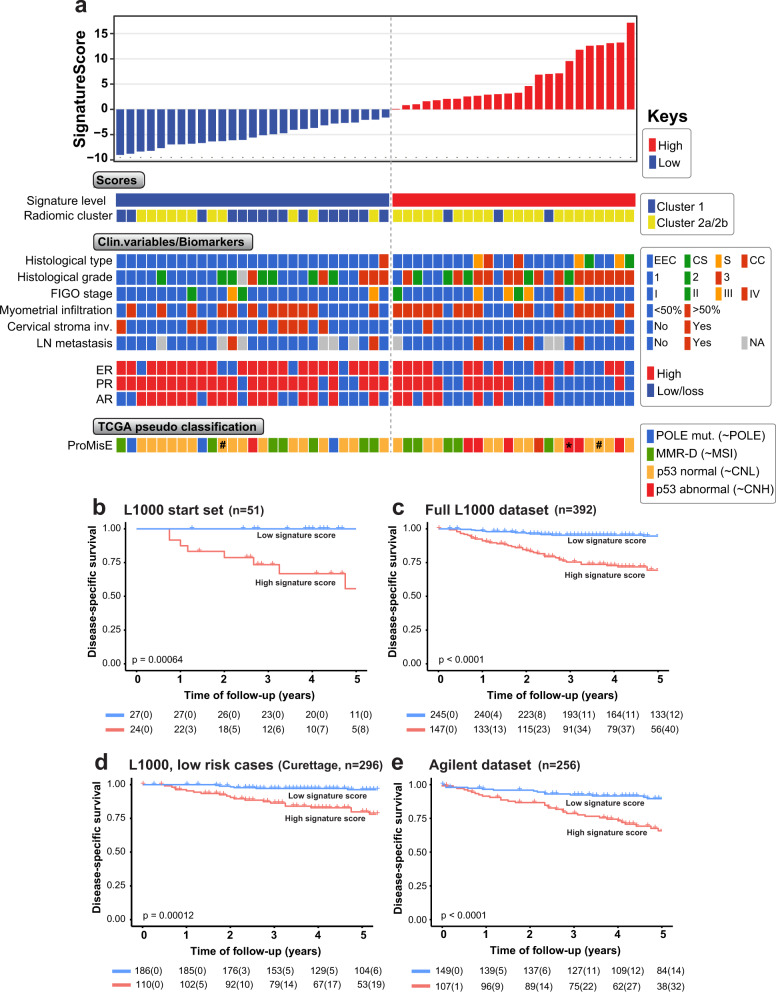
The 11-gene signature score derived from radiomic clusters captures molecular and clinico-pathological patient characteristics and validates in multiple datasets. **a** Top; Ranked 11-gene signature scores calculated on the L1000 gene expression dataset of overlapping patients with manual segmentation-cohort -cohort radiomic data (L1000 start set; *n* = 51 patients). Middle; Clinico-pathological variables and biomarkers panel of protein levels of hormone receptors measured by immunohistochemistry (IHC). Bottom: tentative molecular TCGA class as defined by the ProMisE classifier. Data for the same patient is displayed vertically across panels. **b**–**e** Disease-specific Kaplan–Meier survival analysis of the 11-gene signature in the L1000 start set (**b** *n* = 51 patients), Full L1000 dataset (**c** *n* = 392), L1000 low-risk cases (**d** evaluated by curettage/pipelle indicating endometrioid tumor grade 1 or grade 2, *n* = 296), and Agilent expression dataset (**e** *n* = 256). Note **b** and **e** are subsets of dataset **c**. The number of events in brackets. Mean signature value was used as a cutoff for high or low signature score expression. Hashtag in TCGA pseudo classification (**a**); information extrapolated as non-MSI based on negative POLE, normal p53 staining, and three negative MMRD markers (the fourth marker was not assessed). Asterisk: patient deemed as p53 abnormal despite POLE mutated. Histological types; EEC endometrioid, CS carcinosarcoma, S serous, CC clear cell.

We performed a differential gene expression analysis (significance of microarray analysis; SAM) of the radiomic clusters (comparing clusters 1 to 2b, or all three clusters) from patients with overlapping MRI and transcriptome data (*n* = 51). Eleven genes overlapped between the two analyses and were selected to compute a robust signature of differentially expressed genes for the radiomic clusters. The gene signature included three upregulated genes; *HSPA5*, *GATA3*, and *HSP90AA1*, and eight downregulated genes; *SCGB2A1*, *GSTK1*, *MMP7*, *GDF15*, *ANXA1*, *SAT1*, *CNDP2*, and *PBX1*, related to the most aggressive clusters (Table 2). Patients with tumors of high signature score (cutoff above mean signature score) more often exhibited non-endometrioid histologic type (*p* = 0.019) and tended to be high-grade tumors (*p* = 0.09) with advanced FIGO stage (*p* = 0.23). High signature score patients also had biomarker patterns (Fig. 3a, middle panel) associated with aggressive disease described by loss/low levels of the hormone receptors ER (*p* = 0.037), PR (*p* = 0.005), and AR (*p* = 0.013) (Supplementary Table S2). In addition, substitute TCGA-markers (Fig. 3a) indicated that patients with high signature scores were more often classified as TCGA-copy-number-high (p53 abnormal expression; 87% [7/8 cases]) known to indicate a poor prognosis, and clustered to the radiogenomics cluster 2a/b. Patients with low signature scores were more often of the TCGA equivalent POLE class tumors (3/3 cases) indicating favorable prognosis, while the “MSI”- and “CNL”-like classes were more evenly distributed for the high/low signature score groups. We compared the dichotomous high/low gene expression signature levels with the radiomics clusters (cluster 1 vs. 2a/b) among the 51 samples with overlapping data and found that a significantly higher proportion of patients within cluster 1 had a low signature score (84%; 16/19), compared to that of patients within cluster 2 (34%; 11/32) (*p* = 0.001). The high signature score predicted poor survival in patients with overlapping radiogenomics data (*n* = 51; Fig. 3b; *p* = 0.0006) and the poor prognostic impact of the high signature score was reproduced in the EC cohort with full L1000 gene expression data (*n* = 392) (Table 3, all clinico-pathological variables highly significant; Fig. 3c, *p* < 0.0001). Also, in the subgroup of patients having low-risk histology (endometrioid, grade 1–2) based on preoperative curettage(pipelle)/endometrial biopsy, the 11-gene signature had a prognostic impact with high signature score predicting aggressive clinico-pathological features and poor survival (*n* = 296, Supplementary Table S3 and Fig. 3d; *p* = 0.00012). The association between high signature score and high-risk disease/poor outcome, was also reproduced in the Agilent expression dataset (*n* = 256 patients; Fig. 3e; *p* < 0.0001; Supplementary Table S4) and in an external EC RNA sequencing dataset from TCGA (*n* = 298; Supplementary Fig. S2; *p* = 0.0019). In the latter, a higher proportion of TCGA-copy-number-high (Serous-like) class tumors was also observed in patients with high signature score (49%; 48/97) compared to that of patients with low signature scores (9%; 12/135; *p* < 0.0001).

**Table 2:** List of 11 genes in signature.

Gene name	Signature direction^b^	Entrez gene accession id	Gene description (HGNC Symbol^a^)
*HSPA5*	Up	ENSG00000044574	Heat shock protein family A (Hsp70) member 5
*GATA3*	Up	ENSG00000107485	GATA binding protein 3
*HSP90AA1*	Up	ENSG00000080824	Heat shock protein 90 alpha family class A member 1
*SCGB2A1*	Down	ENSG00000124939	Secretoglobin family 2A member 1
*GSTK1*	Down	ENSG00000197448	Glutathione S-transferase kappa 1
*MMP7*	Down	ENSG00000137673	Matrix metallopeptidase 7
*GDF15*	Down	ENSG00000130513	Growth differentiation factor 15
*ANXA1*	Down	ENSG00000135046	Annexin A1
*SAT1*	Down	ENSG00000130066	Spermidine/spermine N1-acetyltransferase 1
*CNDP2*	Down	ENSG00000133313	Carnosine dipeptidase 2
*PBX1*	Down	ENSG00000185630	PBX homeobox 1

^a^Human Genome Organisation (HUGO) Gene Nomenclature Committee.

^b^Compared to the most aggressive cancers.

**Table 3:** Clinico-pathological variables in relation to the 11-gene signature score in the L1000 dataset (*n* = 392).

Variable	Description	Signature score, *n* (%)		*p* value^a^
		Low (*n* = 245)	High (*n* = 147)	
Age	<66	143 (70.1)	61 (29.9)	0.002
	≥66	102 (54.3)	86 (45.7)	
Histologic type	Endometrioid	238 (75.6)	77 (24.4)	<0.001
	Non-endometrioid	7 (9.1)	70 (90.9)	
Histologic grade^b^	Grade 1–2	212 (83.5)	42 (16.5)	<0.001
	Grade 3	23 (39.7)	35 (60.3)	
FIGO stage	I–II	223 (65.6)	117 (34.4)	0.002
	III–IV	22 (42.3)	30 (57.7)	
Myometrial infiltration	<50%	165 (71.1)	67 (28.9)	<0.001
	≥50%	80 (50.0)	80 (50.0)	
Lymph node metastasis	No	179 (62.2)	109 (37.8)	0.006
	Yes	13 (37.1)	22 (62.9)	
Ploidy	Diploid	148 (69.2)	66 (30.8)	<0.001
	Aneuploidy	20 (30.3)	46 (69.7)	
ER protein expression^c^	High expression	184 (74.5)	63 (25.5)	<0.001
	Low expression	29 (30.2)	67 (69.8)	
PR protein expression^c^	Positive	199 (77.4)	58 (22.6)	<0.001
	Negative	17 (19.3)	71 (80.7)	
AR protein expression^c^	Positive	150 (73.9)	53 (26.1)	<0.001
	Negative	50 (43.9)	64 (56.1)	

Missing data (numbers): Histologic grade (3).

Not assessed (numbers): Lymph node metastasis (69), Ploidy (112), ER (49), PR (47), and AR (75).

^a^Calculated with Chi-Square test or Fischer’s exact test, as appropriate.

^b^Endometrioid type only.

^c^Protein levels by immunohistochemistry (IHC).

### ML-based clustering of patients in the validation cohort confirms the link to poor prognosis

**Fig. 4:**
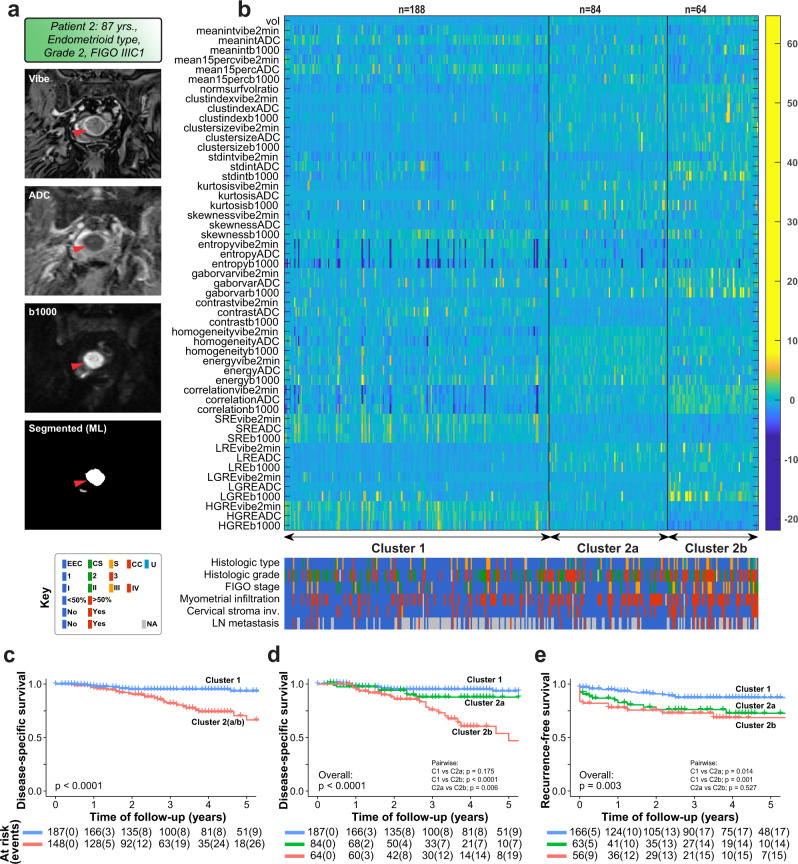
Radiomic clustering of ML segmented tumors in 336 EC patients reproduces three distinct clusters with different prevalence of high-risk features. **a** Preoperative pelvic MRI with automated ML-based tumor segmentation of EC tumor (red arrows) of a patient with endometrioid type, histologic grade 2, and FIGO stage IIIC1. Tumor segmentation was performed by a machine learning (ML) algorithm trained on the segmentation data from the manual segmentation cohort. The radiologic images of the ML-cohort of VIBE + C, ADC, b1000, and tumor segmentation were used for radiomic profiling similar to the manual segmentation-cohort. **b** Heatmap depicting the three distinct radiomic clusters and corresponding clinico-pathologic features capturing different levels of tumor aggressiveness. Patients in cluster 2a (*n* = 84) and 2b (*n* = 64) more often had clinico-pathological variables known to be associated with high-risk disease, compared with that of patients in cluster 1 (*n* = 188). **c**–**e** Survival analysis in relation to the radiomic clusters highlighting a significantly poorer survival for patients in cluster 2. Disease-specific survival comparing clusters 1 and 2a/b combined (**c**), all three clusters (**d**), or by recurrence (**e**). Number of events in brackets. Histological types; EEC endometrioid, CS carcinosarcoma, S serous, CC clear cell, U undifferentiated.

When applying the fully automated machine learning algorithm, the tumor was not detected in 3% (13/349) of the patients and these cases were excluded in subsequent radiomic analyses. For the remaining 336 patients, a 53-feature radiomic cluster analysis was applied using the same algorithm as that for the manually segmented datasets. This yielded clusters comprising 188, 84, and 64 patients in clusters 1, −2a, and −2b, respectively. MRI (VIBE + C, ADC, b1000) with the automated ML-derived tumor segmentation in one representative patient are shown in Fig. 4a. The heatmap of radiomic features for the different clusters (Fig. 4b) showed highly similar discriminating features between the ML clusters to that observed for the manually segmented tumors (Fig. 2b). In agreement with results from the manually segmented dataset, patients in clusters 2a/b more often presented with high-risk clinico-pathological features (Fig. 4b and Supplementary Table S5) and had significantly reduced disease-specific (*p* < 0.0001, Fig. 4c, d) and recurrence-free survival (*p* = 0.003, Fig. 4e). Cluster 2b tumors had higher FIGO stages (*p* = 0.003) and were associated with lymph node metastases (*p* = 0.004), compared to tumors of cluster 2a (Supplementary Table S6).

When comparing the gene expression signature levels across radiomics clusters (clusters 1 and clusters 2a/b) in 98 cases overlapping with the ML-cohort, low signature score tended to be more frequent (74%; 37/50) in patients within cluster 1 compared to that in patients within cluster 2 (56%; 27/48) (*p* = 0.089).

## Discussion

Radiogenomics, linking imaging-, and genomic tumor data, has demonstrated encouraging results in predicting tumor characteristics and survival across many cancer types (reviewed in ref. ^8^). This study, encompassing 866 patients, allowed an unprecedented integrative radiogenomics characterization of EC. Our results show that MRI radiomic tumor profiling identifies distinct radiomic clusters that differentiate between patient groups having significantly different clinico-pathological characteristics and prognosis. Importantly, these radiomic signatures are associated with specific transcriptional programs, suggesting that noninvasive radiomic profiling may aid in assessing patient risk and characterize transcriptional profiles relevant for tumor biology. The identification and validation of distinct radiomic phenotypes emphasize the promising role of radiomic phenotyping as support for developing risk-stratified targeted treatment strategies in EC.

We used two approaches for whole-volume tumor segmentations; manual tumor segmentation (*n* = 138) performed by expert radiologists, and automated deep-learning-based 3D tumor segmentation (*n* = 349). Interestingly, similar radiomic clusters derived from the manual tumor segmentations were reproduced in the radiomic clusters from automated tumor segmentations, and both datasets identified differential transcriptomic signatures, specific clinico-pathological patient characteristics, and different prognosis for the radiomic clusters. For the automated approach, we deployed a convolutional neural network (CNN) deep-learning algorithm allowing expedited whole-volume tumor segmentations, which has been shown to yield tumor segmentations with accuracies comparable to that between radiologists^26^. This finding is supported by the present study suggesting that the approach for automated whole-volume radiomic profiling seems to be valid enough to allow clinical phenotyping and point to likely transcriptional signatures.

The radiomic patient clusters were solely based on radiomic features from the EC tumors without prior feature selection^9^. Radiomic studies report a variable number of features included in their models, presumably related to the imaging modality used, available sequences, and the complexity of the applied radiomic extraction algorithms^7^. We used an approach deriving only 53 features, without subsequent dimensionality reduction. This differs from most studies reporting a multitude of image features with subsequent selection of top features to optimize their models^9,10^. When investigating the interrelationship amongst the 53 features in this study, no single feature was alone able to differentiate between the three clusters (Fig. 2). However, volume-related vectors yielded the longest centroid distance for distinguishing between radiomic cluster 1 and 2b (exhibiting favorable and dismal prognosis, respectively), suggesting a tendency of tumor size to affect clustering. Previously, large tumor size on MRI has been shown to predict deep myometrial invasion, lymph node metastases, high histological grade, advanced FIGO stage, and poor survival in EC, descriptive of aggressive disease^15,22^. We found the radiomic feature “normsurfvolratio” to be independent of tumor volume for tumors with volume >1 ml (*p* = 0.14). Most importantly, this parameter was ranked as the single most important radiomic feature for separating the clusters (Fig. 2d), suggesting that an irregularly shaped tumor is an important predictor of high-risk disease.

We report similar prognostic discrimination by clusters based on dilated and eroded segmentation masks to that based on baseline segmentation masks. Thus, it seems reasonable to conclude that the proposed workflow for feature extraction and clustering is relatively resistant against noise and minor changes of the segmentation masks.

The ML-generated segmentation masks, retrospectively reviewed by an expert radiologist, were judged to include tissue that was likely to represent the primary uterine tumor in as much as 99.7% of the cases (all, except one/336 patients), pinpointing the robustness of our ML-based segmentation method to segment primary tumor tissue in EC.

However, in 29% (96/336) of the patients, the automated ML algorithm yielded more than one segmentation mask, which seemed partly to be caused by heterogeneous tumor growth and partly represent non-tumor tissue in the uterus. In our radiomic analyses we did not exclude any extrauterine segments, but rather included all ML segments unfiltered, aiming to assess whether machine learning-based radiomic signatures were reproduced without any human interventions or adjustments of segmentation masks. Interestingly, this study demonstrates that the developed ML-segmentation algorithm, despite involving segmented areas outside the uterus, is able to capture and reproduce the radiomic features and clusters of clinical relevance in EC.

The 11-gene signature we generated from the radiomic clusters was strongly prognostic and validated in the full L1000 dataset and in orthologous Agilent mRNA expression microarray and external RNA sequencing data from TCGA. Two heat shock proteins (HSPs), HSPA5 and HSP90AA1, were among the three upregulated genes in the signature describing the most aggressive tumors. Increased expression of HSPA5 and HSP90AA1 is in line with previous observations by IHC in EC patients^29,30^. These HSPs can activate the phosphoinositide 3-kinase/protein kinase B (PI3K/AKT)-signaling pathway, the most frequently altered pathway in EC^31^ likely by complexing with PI3K, as experimentally determined cell- and mouse models^32,33^. Interestingly, secreted HSP90α has been shown to induce epithelial-to-mesenchymal transition (EMT) in prostate- cells and tumors^34^, a process that is also activated in aggressive and metastatic endometrial cancer^35^. The third upregulated gene in the signature was the transcriptional factor GATA3, a pioneer transcription factor yet to be functionally described in EC, but that has been linked to the regulation of estrogen signaling in breast cancer^36^. Eight genes in the signature had reduced expression in the aggressive cancers, and decreased expression of SCGB2A1 is known to be associated with poor survival in EC^37,38^. The remaining genes are less characterized in EC tumors but have interesting roles in other cancers related to transcriptional regulation (PBX1 interacts with the estrogen-axis in breast cancer^39^ and SAT1 functions as a transcriptional regulator in aggressive brain tumors^40^), and key signaling pathways including the transforming growth factor β (TGFβ) pathway (GDF15^41^), the PI3K/AKT-pathway (ANX1A^42^), and the Mitogen-activated protein kinase (MAPK) pathway (CNDP2^43^). However, for MMP7, involved in proliferation and metastasis, most studies report elevated expression, suggesting a possible adverse effect of this gene depending on the type of cancer^44^. While the 11-gene signature was driven by differentially expressed genes emerging from clusters 1 and cluster 2b, no genes reached significance when comparing clusters 2a and −2b directly. It is possible that other mechanisms not investigated in this study, such as DNA methylation^45^, could be associated with the radiomic differences between clusters.

The gene signature correlated with the protein expression level of the hormone receptors (ERα, PR, and AR), well known as robust biomarkers in EC^35,46,47^. This finding was consistent across all datasets investigated and emphasizes that the radiomic profiles derived from radiologic image features can capture relevant and well-described biology of the tumors. Interestingly, the distribution of patients is also in accordance with the TCGA classification^31,48^, with POLE positive tumors assigned to the low-risk gene expression signature score, and p53 abnormal tumors mainly found in the high-risk cluster. In a recent publication, a classifier based on contrast-enhanced computed tomography (CE-CT) for the identification of MSI and tumor mutation burden-high (TMB-H) cases was proposed based on a small cohort of endometrial cancers^14^. In this classifier, peritumoral-rim radiomic features were found to be highly important, suggesting that tumors with high mutational burdens have a delineation that can be captured by radiomic profiling^14^. However, the poor soft-tissue resolution at CT often makes valid tumor segmentations difficult, and MRI, yielding much better soft-tissue resolution with an accurate depiction of tumor boundaries, is thus likely to be better suited for whole-volume radiomic profiling. Future radiogenomics studies should evaluate the feasibility of determining the spectrum of TCGA molecular subtypes directly from radiomic data using different imaging modalities in a large cohort.

In the current study, we have demonstrated the translational potential of integrating radiomic profiling with transcriptomic profiling for better preoperative risk assessments in EC patients. While our study has some limitations mainly linked to the retrospective study design with some overlap of cases in the expression datasets, we applied different and independent assays for transcriptome profiling. MRIs were performed using both 1.5 T and 3 T scanners, with possible impact on the radiomic profiles due to systematic differences in signal intensities, and we applied separate Z-transformations for each dataset of the same field strength to account for this. Despite these limitations, we firstly describe distinct radiomic clusters comprising patient groups with differential risk profiles. Secondly, we link these radiomic clusters to differential gene expression and present a gene signature score based on these genes that predicts aggressive features and poor outcome. Thirdly, we demonstrate the feasibility of automated ML-based tumor segmentations for expedited radiomic profiling and clinical phenotyping in EC. Prospective validation in larger and independent patient cohorts should inform potential implementation in the clinic to enable better prognostication and tailoring of treatment in EC.

## Methods

### Patient cohort and biospecimen collection

The current study was conducted under Institutional Review Board (IRB)-approved protocols (2015/2333, 2015/548) and biobank approval (2014/1907) with written informed consent from all patients. Patients were diagnosed and treated at the same University Hospital (Haukeland University Hospital, Bergen, Norway), which is a European Society for Gynecologic Oncology (ESGO) accredited cancer center serving a population of ~1 million inhabitants. Patients diagnosed with histologically confirmed EC during April 2009–July 2019 who had contrast-enhanced MRI performed preoperatively were included and divided into training and a validation cohort as described below. Two radiologists, each with more than 5 years of relevant clinical experience reported on imaging variables and segmented the primary tumors. Clinical data were collected retrospectively from medical records. Expert pathologists evaluated the resected tumors, and tumor cellularity was quantified from hematoxylin- and eosin-stained sections. Preoperative endometrial biopsies by curettage or pipelle were classified as “low risk” from preoperative pathology finding consistent with endometrioid grade 1 or 2 tumors. Biopsies were included if tumor content was more than 70%. RNA was extracted from fresh frozen tissues using Qiagen RNA easy kit (Hilden, Germany) according to the manufacturer’s instructions.

### Imaging protocol and preprocessing steps

Preoperative MRI was performed on a 1.5 T Siemens Avanto running Syngo MR B17 (Erlangen, Germany) (*n* = 266) using a six-channel body coil or on a 3 T Siemens Skyra running Syngo MR E11 (Erlangen, Germany) (*n* = 221) using an 18-channel-body-phased-array and a spine-coil (Supplementary Table S7). Prior to imaging, 20 mg butylscopolamine bromide (Buscopan, Boehringer Ingelheim, Germany) was administered intravenously to reduce bowel peristalsis. A contrast-enhanced T1-weighted axial oblique 3D volumetric interpolated breath-hold (VIBE + C) gradient-echo sequence with fat saturation was acquired 2 min after injection of intravenous contrast agent (0.1 mmol gadolinium/kg body weight, Dotarem, Guerbet, France). In addition, a axial oblique diffusion-weighted imaging (DWI) sequence with b-values of 0 and 1000 s/mm^2^ (1.5 T) or 0, 500 s/mm^2^, and 1000 s/mm^2^ (3 T) was acquired (Supplementary Table S7) in addition to standard T2-weighted sequences. All imaging data were read and reported as part of the standard routine clinical workup prior to treatment. DICOM images from T1 VIBE + C and DWI were exported to NIFTI-1 format^49^ using the conversion tool “mri_convert” as part of “FreeSurfer”^50^. Apparent diffusion coefficient maps (ADC) and b1000 images (DWI image for b = 1000) derived from the DWI data were aligned with the T1-weighted contrast-enhanced VIBE + C sequence using ‘FLIRT’ from the FSL package^51^, leaving a total of three image channels for further analysis.

### Training- and validation imaging cohorts

In total, MRI examinations were available for *n* = 487 patients diagnosed during April 2009 to July 2019; all having visible primary tumors confirmed by a radiologist. The MRI data were divided into a training set with manual tumor segmentations (manual segmentation-cohort, *n* = 138) and a validation set with machine learning derived tumor segmentations (ML-segmentation-cohort, *n* = 349). There were no significant differences between patients in the two cohorts in terms of survival (*p* = 0.8, Supplementary Fig. S3), or clinical variables (Supplementary Table S8) except higher recurrence rate in the manually segmented dataset (*p* = 0.015), which is likely due to longer follow-up time in a manually segmented cohort (47.7 compared to 39.2 months in ML-cohort).

Two radiologists having experience with pelvic MRI outlined all primary tumors in 3D on preoperative MRI in the manual segmentation-cohort (1.5 T, *n* = 71; 3 T, *n* = 67). Tumor labeling was conducted on the VIBE + C 2 min post-contrast images on axial oblique (perpendicular to the long axis of the uterus) slices, the boundaries of the primary tumor were manually drawn and filled to become a binary mask. The remaining MRI examinations in the ML cohort (1.5 T, *n* = 195; 3 T, *n* = 154) were used as a validation cohort with machine learning-based automatic tumor segmentation comprising the ML dataset. The automatic tumor segmentation was performed using a previously published 3D UNet architecture available at Github. (https://github.com/ellisdg/3DUnetCNN)^52^. We implemented a python wrapper for this library, facilitating training and prediction of new datasets. All hyperparameters used in the training process are in detail outlined in Hodneland et al.^26^. The same model was used for the current task of segmenting tumors in the ML dataset. Model weights of the trained network along with a python script (predictUNet3D.py) for predicting primary tumor in new and unseen VIBE + contrast 2 min datasets can be downloaded from https://github.com/ehodneland/RadioGenomicsEC. The same repository also contains code for the training of the network, as well as for extraction and clustering of radiomic features. Finally, all patients (*n* = 487) were assigned an MRI tumor mask segmentation, either by a radiologist or by automatic segmentation.

### Extraction of radiomic profiles

Radiomic features were automatically extracted for the three abovementioned image channels from within the tumor masks, giving a complete set of 53 radiomics features (Supplementary Table S9). For each patient, one scalar value was reported per radiomic feature. The number of radiomic features was selected lower than the number of patients to improve the performance of the prediction model. Tumor volume “vol” was computed as the sum of voxels within the tumor mask times the voxel volume.

To explore the extent of surface folding and irregular tumor shape decoupled from tumor size itself, we initially considered tumor surface area and tumor surface area/tumor volume. However, since both these parameters are inherently closely linked to tumor volume, we created the parameter “normsurfvolratio” =  (V/r^3^)/(A/r^2^). In this formula, r is the radius of an imaginary sphere having the same volume V as the tumor. The tumor volume V and the tumor area A were normalized with r^3^ and r^2^, respectively, in order to remove direct dependencies on tumor volume.

The features “clustersize” and “cluster index” arise from a k-means two-group clustering of the image intensities within the tumor. Objects smaller than three voxels were considered noise after clustering and therefore excluded, while the average volume of the remaining objects were measured to become the “clustersize”. The cluster index “clustindex” is the total number of spatially disconnected objects within the two clusters. “Clustersize” and “clustindex” capture random disorder within the tumor. The features “meaning” and “stdint” are mean and the standard deviation within the tumor, respectively. The feature “mean15perc” is the average intensity of the 15% lowest intensity voxels within the tumor. Kurtosis, skewness, and entropy were calculated using “scipy.stats”^53^. A set of Gabor filters was constructed using the function “gabor_kernel” from “skimage.filters” leading to a 16-dimensional filter bank^54^. The filters were convolved with the tumor image using ‘convolve’ from “scipy.ndimage”. The convolved output was normalized by dividing with the tumor image itself, and the summed output was divided by the number of filters. The final Gabor filter value was reported as the variance of the filter outputs.

The 4D GLCM output matrix was averaged over the search angles and offsets, and normalized to a sum of 1, becoming the GLCM matrix $P$. The element ${P}_{{ij}}$ refers to how often a pixel with grayscale intensity value *i* is adjacent to a pixel with intensity value j, applied to the given search angles and offsets. The GLCM variables contrast, homogeneity, energy, and correlation were estimated by the function “graycoprops” applying the formulas in Supplementary Table S9. The gray level run length matrix (GLRLM) was computed using a tailored algorithm^55^. An output matrix summing the filter response of all filtered angles were summed and divided by the number of filters. The GLRLM matrix $P$ was then normalized to a sum of 1. The element ${P}_{{ij}}$ is the number of homogeneous runs of j voxels with intensity i within the mask. The GLRLM variables short-run emphasis (SRE), long-run emphasis (LRE), low gray level run emphasis (LGLRE), and high gray level run emphasis (HGLRE) were computed as in Supplementary Table S9. All GLCM and GLRLM matrices were computed 2D-wise on paraxial images and later averaged over all tumor slices prior to statistical analysis. Finally, each radiomic feature was Z-transformed (i.e., scaling to standard deviation = 1 and translation to zero mean) across the patients within the training cohort. To avoid selection bias in the clustering analysis due to differences in image intensity between the magnetic field strengths, a Z-transform was carried out separately for images from the 1.5 T and 3 T scanners.

### Unsupervised clustering of radiomic features in the training cohort

Unsupervised clustering was applied to all extracted features to group patients into clusters of similar radiomic patterns. Initially, tumors manually segmented generated two groups using unsupervised k-means clustering with a squared Euclidean distance measure applied to the image feature matrix^56^. Patients in the two clusters displayed differences in clinical characteristics predicting clinical phenotype and survival. With the aim to further refine variability in clinical phenotypes present in the high-risk group, we subsequently performed another unsupervised k-means clustering of the cluster exhibiting the most aggressive clinical characteristics leading to two subclusters. This resulted in three distinct clusters, referred to as cluster 1, cluster 2a, and cluster 2b. K-means clustering with random seed selection is hampered by a non-convex optimization problem and may lead to different results upon each execution cycle. Hence, for the sake of reproducibility, we proceeded with k-means clustering-subclustering (2 + 1) instead of k-means (K = 3). The potentially more stable K-medoids algorithm for clustering was also explored and yielded very similar results to that of K-means with similar risk profiles for the three clusters.

We explored the robustness of the segmentations in terms of minor segmentation errors by dilating and eroding the segmentation mask with a 3 × 3 × 3 structural elements of six connectivity on the training dataset. The radiomic features were extracted and used for a k-means clustering into three clusters as described.

### Assignment of patients to clusters in the ML-cohort

In the ML cohort, patients with no detectable tumor on MRI based on the machine learning algorithm were excluded from further analysis of radiomic profiling (13/349). In the remaining 336 patients, based on automatically segmented tumor volumes, the texture features were Z-transformed for normalization. The normalization parameters of scaling and translation derived from the manual segmentation cohort were applied separately for the 1.5 T and 3 T data. In the next step, we assigned previously unseen patients in the ML cohort to the clusters initially generated from the training cohort. The Euclidean distance between the radiomic feature vector and the cluster centroids was computed for each patient in the ML cohort, and each patient was assigned to belong to the closest cluster in terms of computed distance. Image data from each patient was examined by either one of two trained radiologists evaluating the accuracy of the ML-derived segmentation masks. Out of 336 patients, they found one patient where the ML-suggested lesion was not positioned inside the uterus, and therefore likely not representative of a primary tumor. The automated segmentation algorithm suggested more than one lesion in 29% (96/336) patients; all of them had one lesion in the uterus likely to represent primary tumor, and the additional lesions were in most cases located in putative non-malignant tissue in the uterus or in a few cases located in outside the uterus. All masks were included in the further radiomic profiling since the ML-based tumor segmentation was intentionally conducted completely unsupervised without requiring any manual steps by radiologists. The ranking of feature importance for discriminating clusters was based on pairwise inter-distance cluster centroid distances comparing cluster 1 with −2a, cluster 1 with −2b, and cluster 2a with −2b.

### Gene expression profiling

mRNA expression profiles were generated by the L1000 approach^57^ for 392 patients. The L1000 expression data were generated following an algorithm that extrapolates the expression of 978 directly measured (landmark) genes via a method involving ligation-mediated amplification and fluorescent labeling to obtain a transcriptional profile of 12328 genes in the full L1000 dataset^57^. Replicate-collapsed *z*-scores (level-5 data) were used for subsequent L1000 analysis. L1000 data was available for 51 patients in the MRI-training set, and 112 patients in the validation set. In addition, orthologous mRNA expression profiles were available from previous studies using Agilent microarrays (*n* = 256), including data from overlapping patients (*n* = 98). In brief, Agilent expression data were quantile normalized and log2 transformed from the Agilent Whole Human Genome Microarray kit, 44k (Cat.no. G4112F), as previously described^35^. Validation through TCGA mRNA exon sequencing expression data (IlluminaHiSeq_RNASeqV2, log2 [RPKM + 1] normalized), was performed on data queried from the GDC portal https://www.portal.gdc.cancer.gov.

### Differentially expressed genes and the 11-gene signature score

Analysis of differentially expressed genes between radiomic clusters was performed with the siggenes package in R (version 4.0.0). Significance analysis (SAM) was performed based on selected Delta-values and false discovery rates (FDR), on clusters obtained from unsupervised clustering of radiomic feature extracted matrices. The 11-gene signature was constructed based on the most significant and overlapping genes in SAM analysis employing either two clusters (cluster 1 vs. cluster 2b), or all three clusters (Table 2; Supplementary Data 1). No genes reached significance comparing clusters 2a and −2b in the 51-patient L1000 start set. The signature score of the resulting 11 genes was calculated as the sum of upregulated genes minus the sum of downregulated genes from z-normalized individual expression values^35^.

### Molecular biomarkers and molecular classification

We determined biomarker (protein) expression of hormone receptors (Estrogen Receptor Alpha [ERα], Progesterone Receptor [PR], Androgen Receptor [AR]) by immunohistochemical (IHC) on tissue microarray slides, as previously described in refs. ^35,46,47^. Missing hormone receptor status were extrapolated from expression data compared to cases with both IHC evaluation and L1000 data available, using a mean expression for high/positive or loss/negative markers, for the display of panel in Fig. 3 (*n* = 51 cases). We determined the TCGA molecular-like classes by following the algorithm of the Proactive Molecular Risk Classifier for Endometrial Cancer (ProMisE;^48^). This procedure represents a more clinically feasible classifier than the original proposed by the TCGA^31^. The steps in ProMisE are to be applied sequentially. First, the mismatch repair deficiency was determined (MMRD class; loss of either of MSH6, PMS2, MLH1 or MSH2 by IHC;^58^). Second, the exonuclease domain of polymerase-ε was sequenced (Sanger sequencing of POLE exons 9/11/13/14; POLE class^59^;). Third, the status of protein 53 expression was determined (abnormal p53 by IHC [p53 abnormal, representing *TP53* mutations]) or the alternative p53 wt (p53 normal expression [p53 normal]). The resulting ProMisE classification then provides substitution of the molecular classes originally defined as POLE-ultramutators, MSI-hypermutators, copy-number(variant)-low (Endometrioid), and copy-number-high (Serous-like) classes^31^. We identified four cases with POLE mutations at p.P286R (*n* = 2), p.D287E (*n* = 1), and p.S297F (*n* = 1), all within exon 9 in the manually segmented dataset with overlapping L1000 expression data (*n* = 51).

### Statistics

Data were analyzed using SPSS version 25 (SPSS INC., Chicago, IL) or R (4.0.0). The level of statistical significance was set as *p* < 0.05. All reported *p* values were unadjusted and two-sided. Associations between groups were evaluated using the chi-square test for categorical variables or Fisher’s exact test as appropriate. Kaplan–Meier curves and log-rank tests (Mantel–Cox) were applied for comparing disease-specific and recurrence-free survival between clusters or signatures. The date of primary surgery was defined as the entry date, and the date of death specifically due to endometrial cancer was defined as an event for estimation of disease-specific survival. Recurrence was defined as local recurrence/progression or metastases at later time points.

### Reporting summary

Further information on research design is available in the Nature Research Reporting Summary linked to this article.

## Supplementary information

Supplementary Information

Description of Additional Supplementary Files

Supplementary Data 1

Reporting Summary

## Data Availability

Validation data are available from the TCGA database via https://www.portal.gdc.cancer.gov and https://www.cbioportal.org^60,61^. The transcriptome datasets are deposited at ArrayExpress with accession reference E-MTAB-5017^62^ for Agilent microarray data and E-MTAB-10668^63^ for L1000 data, respectively. Other data of this study are available within supplementary files or the corresponding author upon reasonable request and if in compliance with the general data protection regulation (GDPR) and patient consents.

## Code availability

Matlab and python codes for data processing and analysis, and example input data, are available in the Github repository: https://github.com/ehodneland/RadioGenomicsEC and archived in Zenodo 10.5281/zenodo.5617896.
