# Supplementary material for: A radiogenomics application for prognostic profiling of endometrial cancer
Source: Commun Biol. 2021 Dec 6;4:1363. doi: 10.1038/s42003-021-02894-5 (PMC8648740; doi:10.1038/s42003-021-02894-5)
Supplement: Supplementary file 1 — Supplementary Information [file 42003_2021_2894_MOESM1_ESM.pdf]

Supplementary Information

"A radiogenomics application for prognostic profiling of endometrial cancer"

Hoivik, E.A., Hodneland, E. *et al*.

3 Supplementary Figures

9 Supplementary Tables

Supplementary Figure S1

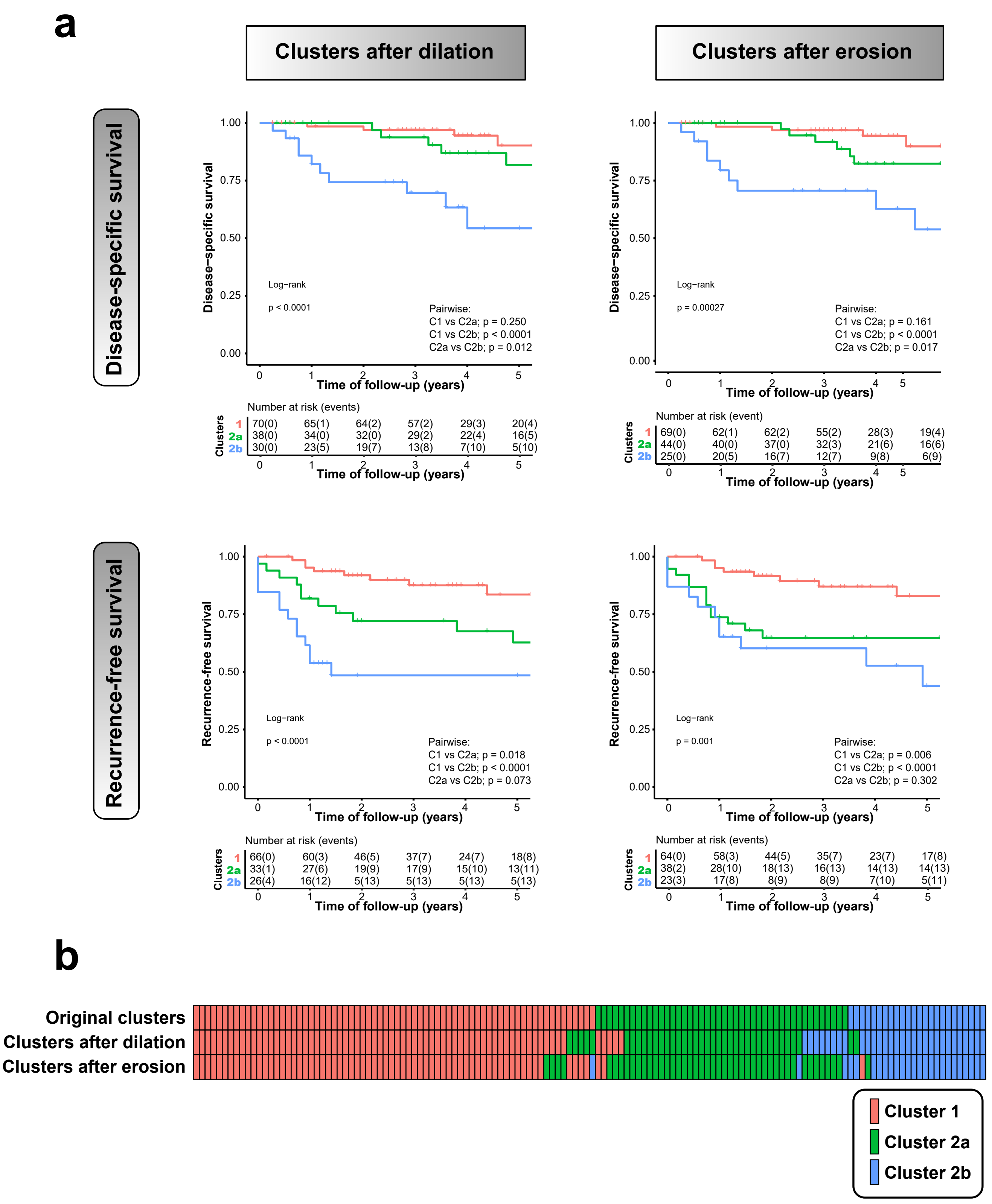

Supplementary Figure S1: Effect of segmentation mask dilation and erosion on survival.
A) Disease-specific survival (top) and recurrence-free survival (bottom) according to radiomic clusters based on dilated segmentation masks (left) and eroded segmentation masks (right). Please see Fig. 2E-F for survival analysis of the original clusters. B) Radiomics clusters after dilation and erosion showing that only 14% (20/138) and 8% (11/138) of the patients switched cluster groups after dilation and erosion, respectively (Original radiomics clusters are from Fig. 2B).

Supplementary Figure S2

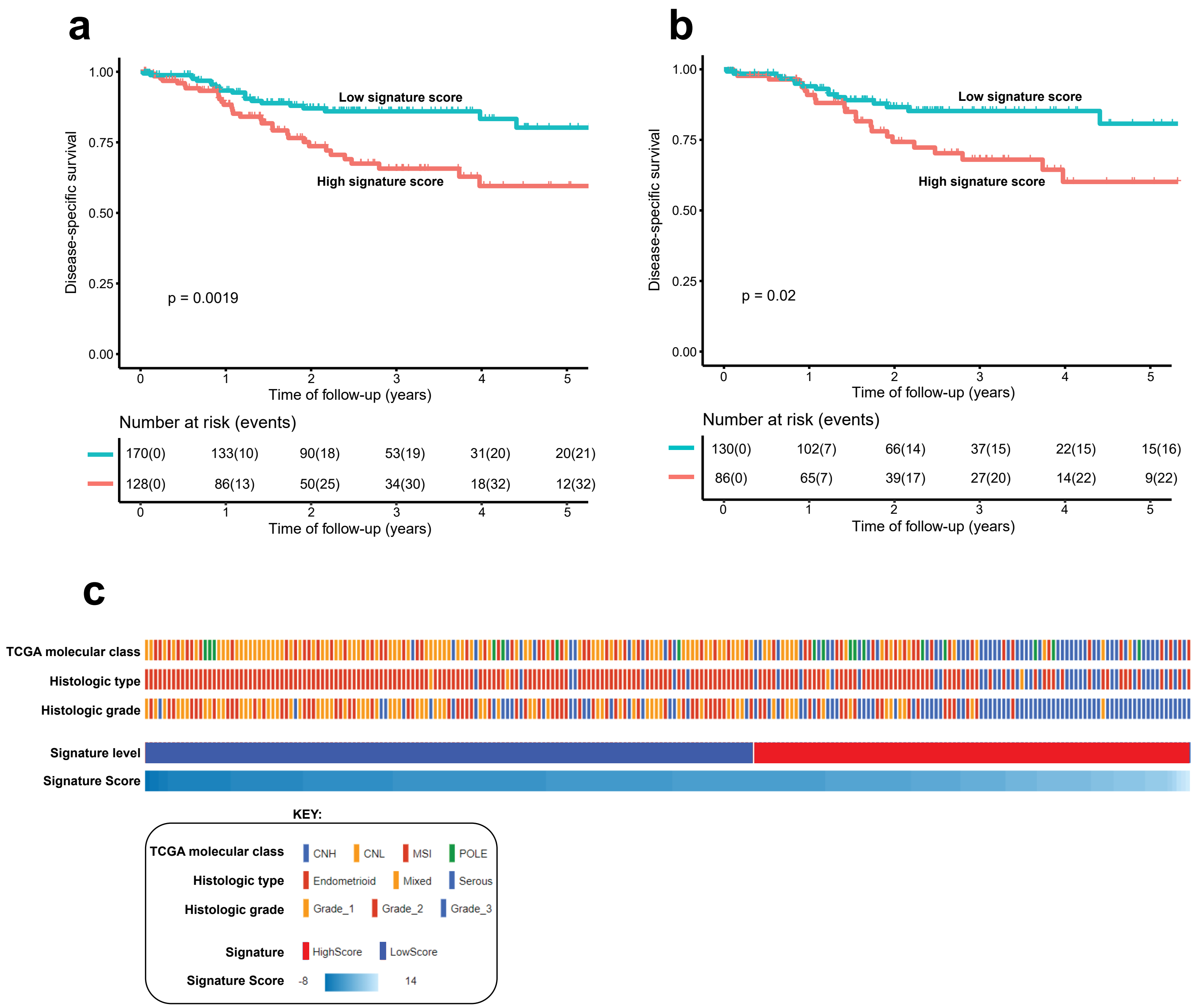

Supplementary Figure S2: Validation of the 11-gene signature score in TCGA RNA sequencing data and in relation to the TCGA molecular classification system. A) Survival by all available samples with UCEC RNA sequencing data and clinical data, n=298. B) Survival with focus on samples with defined TCGA molecular classification, n=232. C) Details of the TCGA molecular class in relation to the 11-gene signature of same patients as displayed in B, ordered by increasing signature score value. The TCGA subclasses consists of; hypermutated (POLE), ultramutators (MSI), copy-number-low (CNL/Endometrioid) and the copy-number-high (CNH/Serous-like) characterized by distinct molecular properties. The TCGA endometrial cancer (UCEC) [1] molecular classes were accessed through https://www.cbioportal.org [2,3].

Supplementary Figure S3

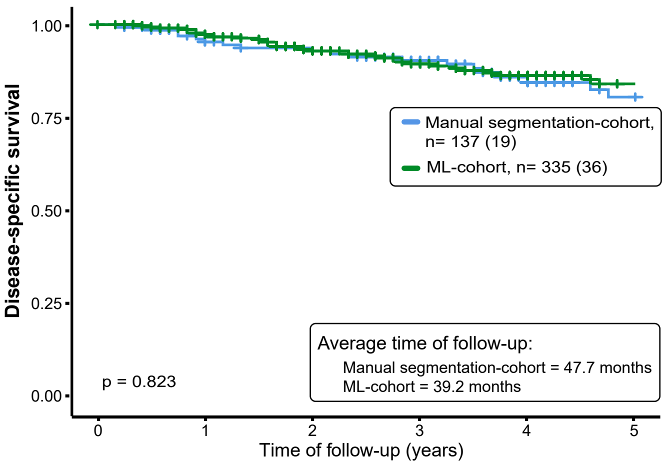

Supplementary Figure S3: Comparison of survival between the manual segmentation-cohort and the ML-cohort. Disease-specific survival between the cohorts demonstrating no differences in survival (p=0.823), although the ML-dataset had shorter follow-up time than the manual segmentation-cohort (39.2 vs 47.7 months, respectively). Number of events in brackets.

Supplementary Table S1: Clinico-pathological variables in relation to radiomic cluster 2a and cluster 2b based on the manually segmented tumors (n=68).

Variable	Description	Radiomic clusters, n (%)		p-value[a]
		Cluster 2a (n=44)	Cluster 2b (n=24)	
**Age**	<66	16 (57.1)	12 (42.9)	0.311
	≥66	28 (70.0)	12 (30.0)	
**Histologic type**	Endometrioid	35 (71.4)	14 (28.6)	**0.050**
	Non-endometrioid	8 (44.4)	10 (55.6)	
**Histologic grade[b]**	Grade 1-2	26 (81.3)	6 (18.8)	**0.050**
	Grade 3	9 (52.9)	8 (47.1)	
**FIGO stage**	I-II	36 (69.2)	16 (30.8)	0.133
	III-IV	7 (46.7)	8 (53.3)	
**Myometrial infiltration**	<50%	10 (55.6)	8 (44.4)	0.401
	≥50%	33 (67.3)	16 (32.7)	
**Lymph node metastasis**	No	26 (61.9)	16 (38.1)	0.105
	Yes	4 (33.3)	8 (66.7)	
**Ploidy**	Diploid	19 (61.3)	12 (38.7)	0.742
	Aneuploidy	7 (53.8)	6 (46.2)	
**ER protein expression[c]**	High expression	18 (75.0)	6 (25.0)	0.111
	Low expression	9 (47.4)	9 (52.6)	
**PR protein expression[c]**	Positive	23 (82.1)	5 (17.9)	**0.001**
	Negative	4 (28.6)	10 (71.4)	
**AR protein expression[c]**	Positive	12 (80.0)	3 (20.0)	0.265
	Negative	10 (58.8)	7 (41.2)	

Missing data (numbers): Histologic type (1), FIGO stage (1), Myometrial infiltration (1).*
Not assessed (numbers): Lymph node metastasis (14), Ploidy (24), ER (25), PR (26) and AR (36).*
[*] *Including one inoperable patient.*
[a] *Calculated with Chi-Square test or Fischer`s exact test, as appropriate.*
[b] *Endometrioid type only.*
[c] *Protein levels by immunohistochemistry (IHC).*

Supplementary Table S2: Clinico-pathological variables in relation to the 11-gene signature score in the L1000 dataset (n=51).

**Variable**	**Description**	**Signature score, n (%)**		**p-value[a]**
		**Low (n=27)**	**High (n=24)**	
**Age**	<66	15 (62.5)	9 (37.5)	0.264
	≥66	12 (44.4)	15 (55.6)	
**Histologic type**	Endometrioid	26 (60.5)	17 (39.5)	**0.019**
	Non-endometrioid	1 (12.5)	7 (87.5)	
**Histologic grade[b]**	Grade 1-2	21 (67.7)	10 (32.3)	0.086
	Grade 3	4 (36.4)	7 (63.6)	
**FIGO stage**	I-II	25 (56.8)	19 (43.2)	0.232
	III-IV	2 (28.6)	5 (71.4)	
**Myometrial infiltration**	<50%	15 (65.2)	8 (34.8)	0.160
	≥50%	12 (42.9)	16 (57.1)	
**Lymph node metastasis**	No	19 (52.8)	17 (47.2)	0.663
	Yes	2 (33.3)	4 (66.7)	
**Ploidy**	Diploid	12 (52.2)	11 (47.8)	0.183
	Aneuploidy	1 (16.7)	5 (83.3)	
**ER protein expression[c]**	High expression	20 (66.7)	10 (33.3)	**0.037**
	Low expression	6 (33.3)	12 (66.7)	
**PR protein expression[c]**	Positive	23 (67.6)	11 (32.4)	**0.005**
	Negative	3 (21.4)	11 (78.6)	
**AR protein expression[c]**	Positive	13 (86.7)	2 (13.3)	**0.013**
	Negative	8 (40.0)	12 (60.0)	

Missing data (numbers): Histologic grade (1).*

Not assessed (numbers): Lymph node metastasis (9), Ploidy (22), ER (3), PR (3) and AR (16).*

* *Including one inoperable patient.*

[a] *Calculated with Chi-Square test or Fischer`s exact test, as appropriate.*

[b] *Endometrioid type only.*

[c] *Protein levels by immunohistochemistry (IHC).*

Supplementary Table S3: Clinico-pathological patient characteristics in relation to the 11-gene signature score in the L1000 dataset in EC patients having preoperative low-risk (endometrioid, grade 1-2) histology based on curettage specimen (n=296).

Variable	Description	Signature score, n (%)		p-value[a]
		Low (n=186)	High (n=110)	
**Age**	<66	109 (66.1)	56 (33.9)	0.226
	≥66	77 (58.8)	54 (41.2)	
**Histologic type**	Endometrioid	186 (66.4)	94 (33.6)	**<0.001**
	Non-endometrioid	0 (0)	16 (100)	
**Histologic grade[b]**	Grade 1-2	168 (70.3)	71 (29.7)	**0.001**
	Grade 3	16 (41.0)	23 (59.0)	
**FIGO stage**	I-II	173 (65.8)	90 (34.2)	**0.004**
	III-IV	13 (39.4)	20 (60.6)	
**Myometrial infiltration**	<50%	127 (70.6)	53 (29.4)	**0.001**
	≥50%	59 (50.9)	57 (49.1)	
**Lymph node metastasis**	No	138 (63.9)	78 (36.1)	**0.004**
	Yes	6 (30.0)	14 (70.0)	
**Ploidy**	Diploid	116 (66.3)	59 (33.7)	**0.037**
	Aneuploidy	13 (44.8)	16 (55.2)	
**ER protein expression[c]**	High expression	148 (70.1)	63 (29.9)	**<0.001**
	Low expression	19 (35.8)	34 (64.2)	
**PR protein expression[c]**	Positive	163 (73.1)	60 (26.9)	**<0.001**
	Negative	6 (14.0)	37 (86.0)	
**AR protein expression[c]**	Positive	123 (71.1)	50 (28.9)	**<0.001**
	Negative	33 (45.2)	40 (54.8)	

Missing data (numbers): Histologic grade (2).
Not assessed (numbers): Lymph node metastasis (60), Ploidy (92), ER (32), PR (30) and AR (50).
[a] *Calculated with Chi-Square test or Fischer`s exact test, as appropriate.*
[b] *Endometrioid type only.*
[c] *Protein levels by immunohistochemistry (IHC).*

Supplementary Table S4: Clinico-pathological variables in relation to the 11-gene signature score in the Agilent dataset (n=256).

Variable	Description	Signature score, n (%)		p-value[a]
		Low (n=149)	High (n=107)	
**Age**	<66	76 (63.9)	43 (36.1)	0.099
	≥66	73 (53.3)	64 (46.7)	
**Histologic type**	Endometrioid	137 (67.5)	68 (32.5)	**<0.001**
	Non-endometrioid	12 (22.6)	41 (77.4)	
**Histologic grade[b]**	Grade 1-2	113 (76.4)	35 (23.6)	**<0.001**
	Grade 3	20 (40.0)	30 (60.0)	
**FIGO stage**	I-II	132 (64.7)	72 (35.3)	**<0.001**
	III-IV	17 (32.7)	35 (67.3)	
**Myometrial infiltration**	<50%	83 (64.3)	46 (35.7)	0.056
	≥50%	65 (52.0)	60 (48.0)	
**Lymph node metastasis**	No	102 (60.7)	66 (39.3)	**0.033**
	Yes	13 (39.4)	20 (60.6)	
**Ploidy**	Diploid	100 (65.8)	52 (34.2)	**<0.001**
	Aneuploidy	11 (28.9)	27 (71.1)	
**ER protein expression[c]**	High expression	122 (69.3)	54 (30.7)	**<0.001**
	Low expression	19 (31.3)	42 (68.9)	
**PR protein expression[c]**	Positive	125 (61.9)	56 (30.9)	**<0.001**
	Negative	18 (30.0)	42 (70.0)	
**AR protein expression[c]**	Positive	81 (66.9)	40 (33.1)	**<0.001**
	Negative	32 (41.0)	46 (59.0)	

Missing data (numbers): Histologic grade (7), Myometrial infiltration (2).
Not assessed (numbers): Lymph node metastasis (55), Ploidy (66), ER (19), PR (15) and AR (57).
[a] *Calculated with Chi-Square test or Fischer`s exact test, as appropriate.*
[b] *Endometrioid type only.*
[c] *Protein levels by immunohistochemistry (IHC).*

Supplementary Table S5: Clinico-pathological variables in relation to the radiomic clusters from automatic tumor (ML) segmentations (n=336).

Variable	Description	Radiomic clusters, n (%)		p-value[a]
		Cluster 1 (n=188)	Cluster 2a/b (n=148)	
**Age**	<66	91 (61.9)	56 (38.1)	0.060
	≥66	97 (51.3)	92 (48.7)	
**Histologic type**	Endometrioid	158 (59.4)	108 (40.6)	**0.010**
	Non-endometrioid	29 (42.0)	40 (58.0)	
**Histologic grade[b]**	Grade 1-2	143 (64.1)	80 (35.9)	**<0.001**
	Grade 3	14 (33.3)	28 (66.7)	
**FIGO stage**	I-II	171 (59.8)	115 (40.2)	**0.001**
	III-IV	17 (34.0)	33 (66.0)	
**Myometrial infiltration**	<50%	131 (66.5)	66 (33.5)	**<0.001**
	≥50%	55 (41.0)	79 (59.0)	
**Lymph node metastasis**	No	95 (53.1)	84 (46.9)	0.237
	Yes	12 (40.0)	18 (60.0)	
**Ploidy**	Diploid	71 (50.0)	71 (50.0)	**0.046**
	Aneuploidy	9 (29.0)	22 (71.0)	
**ER protein expression[c]**	High expression	55 (67.1)	27 (32.9)	0.508
	Low expression	18 (60.0)	12 (40.0)	
**PR protein expression[c]**	Positive	63 (69.2)	28 (30.8)	0.132
	Negative	11 (50.0)	11 (50.0)	
**AR protein expression[c]**	Positive	54 (69.2)	24 (30.8)	0.153
	Negative	21 (55.3)	17 (44.7)	

Missing data (numbers): Histologic type (1), Histologic grade (1), Myometrial infiltration (5).*
Not assessed (numbers): Lymph node metastasis (127), Ploidy (163), ER (224), PR (223) and AR (220).*
* *Including one inoperable patient.*
[a] *Calculated with Chi-Square test or Fischer`s exact test, as appropriate.*
[b] *Endometrioid type only.*
[c] *Protein levels by immunohistochemistry (IHC).*

Supplementary Table S6: Clinico-pathological variables in relation to the radiomic clusters 2a and 2b from automatic tumor (ML) segmentations (n=148).

Variable	Description	Radiomic clusters, n (%)		p-value[a]
		Cluster 2a (n=84)	Cluster 2b (n=64)	
**Age**	<66	36 (64.3)	20 (35.7)	0.173
	≥66	48 (52.2)	44 (47.8)	
**Histologic type**	Endometrioid	66 (61.1)	42 (38.9)	0.094
	Non-endometrioid	18 (45.0)	22 (55.0)	
**Histologic grade[b]**	Grade 1-2	50 (62.5)	30 (37.5)	0.617
	Grade 3	16 (57.1)	12 (42.9)	
**FIGO stage**	I-II	73 (63.5)	42 (36.5)	**0.003**
	III-IV	11 (33.3)	22 (66.7)	
**Myometrial infiltration**	<50%	40 (60.6)	26 (39.4)	0.403
	≥50%	42 (53.2)	37 (46.8)	
**Lymph node metastasis**	No	55 (65.5)	29 (34.5)	**0.004**
	Yes	5 (27.8)	13 (72.2)	
**Ploidy**	Diploid	39 (54.9)	32 (45.1)	0.149
	Aneuploidy	8 (36.4)	14 (63.6)	
**ER protein expression[c]**	High expression	15 (55.6)	12 (44.4)	1.000
	Low expression	6 (50.0)	6 (50.0)	
**PR protein expression[c]**	Positive	15 (53.6)	13 (46.4)	1.000
	Negative	6 (54.5)	5 (45.5)	
**AR protein expression[c]**	Positive	13 (54.2)	11 (45.8)	0.530
	Negative	7 (41.2)	10 (58.8)	

Missing data (numbers): Myometrial infiltration (3).
Not assessed (numbers): Lymph node metastasis (46), Ploidy (55), ER (109), PR (109) and AR (107).
[a] *Calculated with Chi-Square test or Fischer`s exact test, as appropriate.*
[b] *Endometrioid type only.*
[c] *Protein levels by immunohistochemistry (IHC).*

Supplementary Table S7: Scanning protocols used at the 1.5T and 3T MRI scanners.

MR scanner	MR sequence	$TR/TE_1/TE_2$ (ms)	FA (deg)	Slice/Incr. (mm)	Acquisition matrix	Pixel size (mm)
1.5T Siemens Avanto (n=266)	T1 VIBE	7.2/2.6	20	2.0/2.0	192x154	1.3x1.3
	DWI	3100/79	90	5.0/6.0	128x128	2.3x2.3
3T Siemens Skyra (n=221)	T1 DIXON	5.9/2.5/3.7	9	1.2/1.2	139x256	1.0x1.0
	DWI RESOLVE	6010/74/126	180	3.0/3.3	144x144	1.4x1.4

Abbreviations: T=Tesla, TR=repetition time, TE=echo time, FA=flip angle, mm=millimeter, VIBE=volumetric interpolated breath-hold examination, DWI=diffusion weighted imaging, RESOLVE=Readout segmentation of long variable echo trains, Incr.=Increment.

Supplementary Table S8: Comparison of clinico-pathological patient characteristics between manually segmented tumors (n=138) and ML-segmented tumors (n=336).

Variable	Description	Segmentation method, n (%)		p-value[a]
		MANUAL (n=138)	ML (n=336)	
**Age**	<66	66 (47.8)	147 (43.8)	0.477
	≥66	72 (52.2)	189 (56.3)	
**Histologic type**	Endometrioid	112 (81.8)	266 (79.4)	0.613
	Non-endometrioid	25 (18.2)	69 (20.6)	
**Histologic grade[b]**	Grade 1-2	87 (79.8)	223 (84.2)	0.365
	Grade 3	22 (20.2)	42 (15.8)	
**FIGO stage**	I-II	118 (86.1)	286 (85.1)	0.886
	III-IV	19 (13.9)	50 (14.9)	
**Myometrial infiltration**	<50%	74 (54.0)	197 (59.5)	0.304
	≥50%	63 (46.0)	134 (40.5)	
**Lymph node metastasis**	No	82 (85.4)	179 (85.6)	1.000
	Yes	14 (14.6)	30 (14.4)	
**Ploidy**	Diploid	55 (74.3)	142 (82.1)	0.171
	Aneuploidy	19 (25.7)	31 (17.9)	
**ER protein expression[c]**	High expression	51 (65.4)	82 (73.2)	0.263
	Low expression	27 (34.6)	30 (26.8)	
**PR protein expression[c]**	Positive	59 (75.6)	91 (80.5)	0.475
	Negative	19 (24.4)	22 (19.5)	
**AR protein expression[c]**	Positive	31 (51.7)	78 (67.2)	0.050
	Negative	29 (48.3)	38 (32.8)	

Missing data (numbers): Histologic type (2), Histologic grade (4), FIGO stage (1), Menopause (1), Parity (4), Myometrial infiltration (6).

Not assessed (numbers): Lymph node metastasis (169), Ploidy (227), ER (289), PR (288), AR (298), GR (294).

[a] *Calculated with Chi-Square test or Fischer`s exact test, as appropriate.*

[b] *Endometrioid type only.*

[c] *Protein levels by immunohistochemistry (IHC).*

Supplementary Table S9: Overview of radiomic MR features used in the clustering.

Type	Radiomic feature	Abbreviation	Definitions
Morphological	Tumor volume	vol	Volume in ml
	Normalized surface to volume ratio	normsurfvolratio	$(S/r^2)/(V/r^3)$
	Cluster index	clustindex	No. disconnected cluster objects
	Cluster size	clustersize	Average size of cluster objects
Statistical	Mean intensity	meanint	Average image intensity
	Mean intensity 15%	mean15perc	Average image intensity lower 15% percentile
	Standard deviation	stdint	Standard deviation
	Kurtosis	kurtosis	Fourth standardized moment
	Skewness	skewness	Third standardized moment
	Entropy	entropy	30 bins in histogram
	Gabor filter	gaborvar	Variance of 16-dimensional filter output; angles=[0, π/4, π/2, 3π/4] rad, standard deviations=[1,3] vox, and frequencies = [0.05, 0.25] vox^{-1}
Structural	Contrast*	contrast	$\sum_{i,j} p_{ij}(i-j)^2$
	Homogeneity*	homogeneity	$\sum_{i,j} p_{ij}/(1+(i-j)^2)$
	Energy*	energy	$\sqrt{\sum_{i,j} {p_{ij}}^2}$
	Correlation*	correlation	$\sum_{i,j} p_{ij}\left((i-\mu_i)(j-\mu_j)/\sqrt{\sigma_i^2\sigma_j^2}\right)$
	Short run emphasis°	SRE	$(1/N)\sum_{i,j} p_{ij}/j^2$
	Long run emphasis°	LRE	$(1/N)\sum_{i,j} p_{ij}\cdot j^2$
	Low Gray Level Run Emphasis°	LGRE	$(1/N)\sum_{i,j} p_{ij}/i^2$
	High Gray Level Run Emphasis°	HGRE	$(1/N)\sum_{i,j} p_{ij}\cdot i^2$

Note: Apart from tumor volume and normalized surface to volume ratio, all radiomic features were computed for each of the image MR channels.

** GLCM feature: GLCM matrix was computed with 'scikit-learn' function 'greycomatrix' with search angles = [0, π/4, π/2, 3 π/4] rad, offset = [1, 3, 5] voxels, number of levels = 24 (Reference [4]).*

° GLRLM features: GLRLM matrix computed with search angles = [0, π/4, π/2, 3 π/4] rad, and 8 grey intervals; P: GLCM or GLRLM matrix; N = total number of homogenous runs within mask for GLRLM matrix; $\mu_{i,j}$ and $\sigma_{i,j}$ = mean and standard deviation of a pixel value in row or column direction of the GLCM matrix.

Supplementary References

1. Cancer Genome Atlas Research Network, Kandoth, C. *et al.* Integrated genomic characterization of endometrial carcinoma. *Nature*. **497**, 67-73 (2013).
2. Cerami, E., *et al.* The cBio cancer genomics portal: an open platform for exploring multidimensional cancer genomics data. *Cancer Discov.* **2**, 401-4 (2012).
3. Gao, J. *et al.* Integrative analysis of complex cancer genomics and clinical profiles using the cBioPortal. *Sci Signal.* **6**, 269 (2013).
4. Pedregosa, F. *et al.* Scikit-Learn: Machine Learning in Python. *Journal of Machine Learning Research*, **12**, 2825-2830 (2011).
