# Supplementary material for: A radiogenomics application for prognostic profiling of endometrial cancer
Source: Commun Biol. 2021 Dec 6;4:1363. doi: 10.1038/s42003-021-02894-5 (PMC8648740; doi:10.1038/s42003-021-02894-5)
Supplement: Supplementary file 2 — Description of Additional Supplementary Files [file 42003_2021_2894_MOESM2_ESM.pdf]

Description of Additional Supplementary Files

File name: Supplementary Data 1.
Description: SAM analysis.
