# Supplementary material for: A radiogenomics application for prognostic profiling of endometrial cancer
Source: Commun Biol. 2021 Dec 6;4:1363. doi: 10.1038/s42003-021-02894-5 (PMC8648740; doi:10.1038/s42003-021-02894-5)
Supplement: Supplementary file 4 — Reporting Summary [file 42003_2021_2894_MOESM4_ESM.pdf]

nature research

Corresponding author(s): Hoivik, Haldorsen, Krakstad

Last updated by author(s): October 04., 2021

Reporting Summary

Nature Research wishes to improve the reproducibility of the work that we publish. This form provides structure for consistency and transparency in reporting. For further information on Nature Research policies, see our Editorial Policies and the Editorial Policy Checklist.

Statistics

For all statistical analyses, confirm that the following items are present in the figure legend, table legend, main text, or Methods section.

n/a	Confirmed	
☐	☒	The exact sample size (*n*) for each experimental group/condition, given as a discrete number and unit of measurement
☐	☒	A statement on whether measurements were taken from distinct samples or whether the same sample was measured repeatedly
☐	☒	The statistical test(s) used AND whether they are one- or two-sided<br>*Only common tests should be described solely by name; describe more complex techniques in the Methods section.*
☐	☒	A description of all covariates tested
☐	☒	A description of any assumptions or corrections, such as tests of normality and adjustment for multiple comparisons
☒	☐	A full description of the statistical parameters including central tendency (e.g. means) or other basic estimates (e.g. regression coefficient) AND variation (e.g. standard deviation) or associated estimates of uncertainty (e.g. confidence intervals)
☐	☒	For null hypothesis testing, the test statistic (e.g. *F*, *t*, *r*) with confidence intervals, effect sizes, degrees of freedom and *P* value noted<br>*Give P values as exact values whenever suitable.*
☒	☐	For Bayesian analysis, information on the choice of priors and Markov chain Monte Carlo settings
☒	☐	For hierarchical and complex designs, identification of the appropriate level for tests and full reporting of outcomes
☐	☒	Estimates of effect sizes (e.g. Cohen's *d*, Pearson's *r*), indicating how they were calculated

Our web collection on statistics for biologists contains articles on many of the points above.

Software and code

Policy information about availability of computer code

Data collection	MRI instruments; 1.5T Siemens Avanto running Syngo MR B17 and 3T Siemens Skyra running Syngo MR E11 (Erlangen, Germany)
Data analysis	Python (3.6), MATLAB (R2020b), R (4.0.0) , SPSS (25). Matlab and python codes for data processing and analysis and example data input are available in the Github repository: https://github.com/ehodneland/RadioGenomicsEC

For manuscripts utilizing custom algorithms or software that are central to the research but not yet described in published literature, software must be made available to editors and reviewers. We strongly encourage code deposition in a community repository (e.g. GitHub). See the Nature Research guidelines for submitting code & software for further information.

Data

Policy information about availability of data

All manuscripts must include a data availability statement. This statement should provide the following information, where applicable:

- Accession codes, unique identifiers, or web links for publicly available datasets
- A list of figures that have associated raw data
- A description of any restrictions on data availability

Validation data are available from the TCGA database via https://www.portal.gdc.cancer.gov and https://www.cbioportal.org 58,59. The transcriptome datasets are deposited at ArrayExpress with accession reference E-MTAB-5017 for Agilent microarray data and E-MTAB-10668 for L1000 data, respectively. Other data of this study are available within supplementary files or the corresponding author upon reasonable request and if in compliance with the general data protection regulation (GDPR) and patient consent.

Field-specific reporting

Please select the one below that is the best fit for your research. If you are not sure, read the appropriate sections before making your selection.

☒ Life sciences ☐ Behavioural & social sciences ☐ Ecological, evolutionary & environmental sciences

For a reference copy of the document with all sections, see nature.com/documents/nr-reporting-summary-flat.pdf

Life sciences study design

All studies must disclose on these points even when the disclosure is negative.

Sample size	No sample-size calculation was performed due to study design.
Data exclusions	13/349 cases was excluded as no tumor was detected during ML-based segmentation.
Replication	All findings were reproduced and validated successfully in additional datasets.
Randomization	Cases were randomly assigned to manual or ML-cohorts. Clinical variables and survival was compared, with no differences.
Blinding	Blinding not relevant as ML-application was involved.

Reporting for specific materials, systems and methods

We require information from authors about some types of materials, experimental systems and methods used in many studies. Here, indicate whether each material, system or method listed is relevant to your study. If you are not sure if a list item applies to your research, read the appropriate section before selecting a response.

Materials & experimental systems

n/a	Involved in the study	
☒	☐	Antibodies
☒	☐	Eukaryotic cell lines
☒	☐	Palaeontology and archaeology
☒	☐	Animals and other organisms
☒	☐	Human research participants
☐	☒	Clinical data
☒	☐	Dual use research of concern

Methods

n/a	Involved in the study	
☒	☐	ChIP-seq
☒	☐	Flow cytometry
☒	☐	MRI-based neuroimaging

Clinical data

Policy information about clinical studies

All manuscripts should comply with the ICMJE guidelines for publication of clinical research and a completed CONSORT checklist must be included with all submissions.

Clinical trial registration	*Provide the trial registration number from ClinicalTrials.gov or an equivalent agency.*
Study protocol	Standardized hospital MR imaging protocol.
Data collection	Haukeland University Hospital, prospectively, April 2009 to July 2019.
Outcomes	Data from medical records, disease-specific survival and clinico-pathological variables.
